# The Effect of lnterleukin-6 Gene Polymorphism on Pediatric Pneumonia

**Published:** 2019-11

**Authors:** Guode SU, Lili DING, Zhenkun ZHANG

**Affiliations:** 1. Department II of Pediatric Respiration, Xuzhou Children Hospital, Xuzhou Medical University, Xuzhou, P.R. China; 2. Department of Neonatology, Xuzhou Children Hospital, Xuzhou Medical University, Xuzhou, P.R. China

**Keywords:** Interleukin-6, Gene polymorphism, Pediatric pneumonia, Community acquired pneumonia

## Abstract

**Background:**

To investigate the effect of interleukin-6 (IL-6) gene polymorphism on pediatric pneumonia.

**Methods:**

Overall, 438 patients with pediatric pneumonia (Observation group) treated in Xuzhou Children Hospital, Cinna from July 2013 to July 2018 were randomly enrolled. Meanwhile, 423 healthy children (Control group) in the same time period were randomly selected. PCR was applied to amplify the IL-6-572 gene fragment. The IL-6-572 polymorphism was detected, and the impacts of gene polymorphism difference on pediatric pneumonia were observed.

**Results:**

There were differences in the IL-6 genotypes between the two groups (*P*<0.05). Among the CG+GG genotypes in Observation group, G allele frequency was higher than that in control group (*P*<0.05). The risk of pediatric pneumonia for GC genotype was 2.13 times as high as that for CC genotype, and the risk of pediatric pneumonia for GG genotype was 5.56 times as high as that for CC genotype.

**Conclusion:**

IL-6 gene polymorphism might be related to the pediatric pneumonia and the population with G allele at this locus may be more prone to pediatric pneumonia.

## Introduction

Pediatric pneumonia is an infectious disease attached great importance to by the pediatric department. Streptococcus pneumoniae, haemophilus influenzae and staphylococcus aureus are the main causes of bacterial pneumonia (1), and that respiratory syncytial virus, parainfluenza virus and influenza virus are the main causes of pediatric viral pneumonia (2). Community acquired pneumonia (CAP) is the leading cause of children’s mortality around the globe (3, 4), with more than 150,000 children requiring hospitalization each year. It is the fifth largest epidemic disease in all pediatric hospitalizations in the United States which has a very high diagnosis value (5).

In August 2011, the Pediatric Infectious Diseases Society (PIDS) and the Infectious Diseases Society of America (IDSA) issued an evidence-based guideline for managing children's CAP. The guideline suggests that the CAP children patients with full immunization and without potential complications shall be hospitalized for observation and receive treatment with aminopenicillin (6). Interleukin-6 (IL-6) is now considered as an important target for clinical intervention. However, signal pathway that Controls IL-6 activity is complex and different intervention strategies can inhibit this pathway (7, 8).

In studies on IL-6 on basis of single region and fragment, the etiology of diseases and all the clinical explanations except for inflammatory markers are not very clear. Therefore, in this study, the impact of IL-6 gene polymorphism on pediatric pneumonia was examined, hoping to find pediatric pneumonia screening indicators and to conduct better prevention and treatment of pediatric pneumonia.

## Materials and Methods

### Methods

A total of 438 patients with pediatric pneumonia (Observation group) and 423 healthy subjects (Control group) diagnosed in Xuzhou Children Hospital, China from July 2013 to July 2018 were randomly selected. Inclusion criteria: 1) All children patients diagnosed with CAP (7), 2) children patients aged less than 12 years old, 3) children patients with normal thyroid function, and 4) children patients without lymphocytosis and positive acid-fast bacilli in the sputum smears. Exclusion criteria: 1) Children patients with diabetes mellitus, 2) children patients with metabolic diseases, 3) children patients with respiratory tract infection, 4) children patients with benign or malignant tumor before or during hospitalization, or 5) children patients with autoimmune diseases. This study was approved by the Ethics Committee of Xuzhou Children Hospital, and the families of the children patients signed the written informed Consent.

Extraction of IL-6 deoxyribonucleic acid (DNA): Genomic DNA was extracted from the whole blood anti-coagulated with ethylene diamine tetraacetic acid (EDTA) according to the TIANamp Genomic DNA kit instruction offered by Thermo Fisher Scientific (China) Co, Ltd. The DNA purity was measured respectively at wave lengths of 260 nm and 280 nm with an ultraviolet spectrophotometer, and the extracted DNA purity was considered to meet the requirements if the optical density (*OD*) *_260_/OD_ 280_* value was 1.7–1.9. It was stored at −20 °C.

Twenty five μL polymerase chain reaction (PCR) system was pre-denatured for 5min at 95°C, denatured for 3min at 95°C, annealed for 30s at 60 °C and extended for 1min at 72 °C. The above mentioned processes constituted one circulation, and 35 circulations were performed. After that, the extension for 7min at 72 °C was performed. Forward primer: 5′-GTTCTACAACAGCCCCTCACAGGGAGAGCC-3′, reverse primer: 5′-GGCTCTCCCTGTGAGGGGCTGTTGTAGAAC-3′. U6 was used as the internal reference, forward primer: 5′-CTCGCTTCGGCAGCACA-3′, reverse primer: 5′-AACGCTTCACGAATTTGCGT-3′. Twenty μL system was enzyme digested at 36°C for 18–24h. The electrophoresis was conducted for the enzyme digestion products by means of 3% agarose gel electrophoresis, and the gel imaging system was used for analysis.

TIANamp Genomic DNA kit was purchased from Thermo Fisher Scientific (China) Co, Ltd., DNA amplification kit from Sigma-Aldrich, MBI kit from Beijing Bio-Rad Life Science Development Co., Ltd., U6 internal reference primer from Guangzhou Shangeng Biotechnology Co., Ltd., agarose from Thermo Fisher Scientific (China) Co, Ltd., UV-9000S type double-beam UV-spectrophotometer from Nanjing Feile Instrument Co., Ltd., PCR amplifier from hermo Fisher Scientific (China) Co, Ltd., and gel imaging analyzer from Beijing Maisiqi High-tech Co., Ltd.

### Statistical analysis

Statistical Package for Social Sciences (SPSS) 19.0 (Chicago, IL, USA) was adopted, and χ^2^ test was applied for all rate comparisons. Measurement data were expressed by *x̄*±*s*, and non-parametric Kolmogorov-Smirnov (K-S) test was used for analysis. The correlation between IL-6 genotype and the risk of pediatric pneumonia in children patients was analyzed through COX regression analysis. *P*<0.05 suggested significant difference.

## Results

### Clinical data

Among the 438 patients with pediatric pneumonia (Observation group) diagnosed in Xuzhou Children Hospital from July 2013 to July 2018, there were 197 male patients and 241 female patients with an average age of 5.6 (2.4) years old and a course of disease of 11.4 (4.3) days. Among the children in control group, there were 215 males and 208 females with an average age of 6.3 (2.9) years old. There were no statistical differences in gender and age between the two groups of children patients, while there were statistical differences in heart rate, respiratory rate, blood oxygen saturation, white blood cell count and C-reactive protein (CRP) between the two groups of children patients (*P*<0.05). The children patients in Observation group had higher heart rate, respiratory rate, white blood cell count and CRP (*P*<0.05), but lower blood oxygen saturation (*P*<0.05), than Control group (Table 1).

**Table 1:** Comparisons of clinical data between the two groups of children patients

***Variable***	***Control group (n=423)***	***Observation group (n=438)***	**P**
Gender (male/female)	215/208	197/241	0.526
Age (yr)	6.3 (2.9)	5.6 (2.4)	0.738
Course of disease (day)		11.4 (4.3)	
Heart rate	95.3 (5.6)	166.6 (7.5)	0.031
Respiratory rate	30.2 (3.7)	63.4 (4.1)	0.022
Blood oxygen saturation	98.5 (2.4)	85.5 (4.9)	0.032
White blood cell count	6.4 (1.3)	13.6 (2.8)	0.015
CRP	4.3(3.2)	37.6(2.8)	0.001

### Hardy-Weinberg equilibrium analysis

The genotypic frequencies at the single nucleotide polymorphism (SNP) (IL-6-572) locus of two genes were counted through Genetics package analysis. The IL-6-572 gene frequencies in both Control group and patient group conformed to the Hardy-Weinberg law, indicating that this study is representative (Table 2).

**Table 2:** Hardy-Weinberg equilibrium analysis

***Gene locus***	***Control group (n=423)***		***Observation group (n=438)***	
IL-6-572	*χ^2^*	P	*χ^2^*	P
	0.732	0.411	0.515	0.514

### MbiI restriction enzyme could cut the position in IL-6-572 where C was replaced by G

After digestion, 3 genotypes were detected in IL-6-572, namely CC type with 163 bp, GG type with 101 bp and 62 bp, and CG type with 163 bp, 101 bp and 62 bp. Random sampling was performed by Guangzhou Shangeng Biotechnology Co., Ltd. for gene sequencing, and it was found that the two inspection results were consistent.

### PCR method was applied to amplify the IL-6-572 gene fragment

The IL-6 genotypes in the two groups of children patients included GG type, GC type and CC type. In Observation group, there were 39 cases of GG type, 193 cases of GC type and 206 cases of CC type. Among children in Control group, there were 14 cases of GG type, 58 cases of GC type and 351 cases of CC type. There existed genotype differences between the two groups (*P*=0.038, 0.021, 0.032). Among the CG+GG genotypes in Observation group, the G allele frequency was higher than that in control group (*P*<0.05) (Table 3).

**Table 3:** Analysis of genotype between the two groups of children patients

***Variable***	***Control group (n=423)***	***Observation group (n=438)***	**P**
GG	14	39	0.038
GC	58	193	0.021
CC	351	206	0.032
C	87.8 %	721 %	0.006
G	12.2 %	27.9 %	-

### The genotype frequency and relative risk of pediatric pneumonia were compared

The results showed that the risk of pediatric pneumonia for GC genotype was 2.1 times as high as that for CC genotype [95% confidence interval (95% CI): 1.0–4.2], and the risk of pediatric pneumonia for GG genotype was 5.6 times as high as that for CC genotype (95% CI: 1.2–24.8).

## Discussion

CAP in children is a worldwide problem (8, 9). More than 500,000 CAP children were treated in the emergency department in the United States, accounting for 7% of hospitalized children patients in the pediatric department per year (10, 11). IL-6 is widely present in various inflammatory responses, and therapeutic efficacy of blocking IL-6 has been proven in Castleman disease and inflammatory diseases (rheumatoid arthritis) (12). IL-6 was important for the 30-day mortality prediction of the hospitalized CAP patients (13).

In this study, the IL-6 gene polymorphism in the CAP children patients was analyzed and the correlation between them was explored. The Hardy-Weinberg equilibrium analysis was conducted first, and the results manifested that the gene frequency was not different between the two groups of children patients.

Moreover, the analysis on the basic data also revealed that there were no statistical differences in gender and age between the two groups of children patients, implying that this study was comparable. According to this study, the IL-6-572 gene fragment was amplified via PCR, and there were differences in the IL-6 genotypes (GG type, GC type and CC type) between the two groups of children patients. Further analysis indicated that the frequency of G allele in IL-6-572 C/G gene fragment in the CAP children patients was significantly higher than that in healthy children. A fragment of IL-6 was studied and the result suggested that IL-6-572 G allele may be a risk factor for the CAP. Since this study only focused on single region and fragment; namely, SNP of IL-6 gene, the correlations of different loci with the onset risk of pediatric pneumonia need to be further investigated, so the results are only for reference. Zidan et al (14) studied the impact of IL6-174G/C on the CAP children, and the result suggested that the G allele in IL6-174G/C and the CAP susceptibility were closely related. Although this study researched different fragments of IL-6, consistent results were obtained that the two gene fragments might have consistent effect in the CAP. However, based on the results of this study, the degree of effect of these two gene fragments in the CAP could not be predicted. Nevertheless, it can be used as a future research direction, including which part of the CAP possibly affected by the two gene fragments.

The frequency of A allele in IL-6-597G/A (AA+AG) genotype of CAP patients was significantly higher than that of healthy population (*P*<0.05) (15). They argued that A allele may be a risk factor for the CAP patients, but they did not discover a significant relevance between IL-6-572C/G (rs1800796) and the CAP. The difference between their and our results suggests that the role of G allele is different in IL-6-572C/G and IL-6-597G/A, or that G allele in IL-6-597G/A has less significant effect in CAP than A allele. Therefore, the role of G allele in IL-6-597G/A in other diseases may be explored in the future, and the relationship between IL-6-572C/G and the severity of CAP can be further studied.

IL-6 gene is a very important gene, whose role has been found in many diseases, including tumors, through the current studies on IL-6 gene polymorphism. Carriers of G allele in IL6-174 have less depressive symptoms after interpersonal stress, but the IL6 genotype does not alleviate the impacts of non-interpersonal stress exposure (i.e., financial, work and health-related difficulties) on depression (16). IL-6-597A/G and IL-6-174G/C are closely correlated with type 2 diabetes susceptibility (17). IL-6-174G/C polymorphism has a close association with cancer pain of patients with non-small cell lung cancer (18).

IL-6 is also a therapeutic target for many diseases, and the efficacy of anti-IL-6 receptor (IL-6R) Tocilizumab as a single therapy or in combination with anti-rheumatic drugs in the treatment of moderate to severe rheumatoid arthritis in adults has been demonstrated (19). Anti-IL-6 target therapy can significantly improve the symptoms of systemic sclerosis (20). IL-6 may also serve as a therapeutic target for pediatric pneumonia, and researchers can conduct a further study in the future.

## Conclusion

IL-6 gene polymorphism may be associated with pediatric pneumonia, and the population carrying G allele at that locus may be more prone to pediatric pneumonia. IL-6 gene can be used as a screening index for pediatric pneumonia, so as to assist in the prevention and treatment of pediatric pneumonia.

## Ethical considerations

Ethical issues (Including plagiarism, informed consent, misconduct, data fabrication and/or falsification, double publication and/or submission, redundancy, etc.) have been completely observed by the authors.
